# Supplementary material for: Deletion of wfs1 Impairs Oligodendrocyte Precursor Cells Dorsal Distribution and Myelination Through the wfs1-hmgcs1 Axis in Zebrafish
Source: Biology (Basel). 2026 Aug 16;15(16):1402. doi: 10.3390/biology15161402 (PMC13510151; doi:10.3390/biology15161402)
Supplement: Supplementary file 1 [file biology-15-01402-s001.zip › File S1-Transcriptome sequencing data and differentially expressed genes (DEGs) in wfs1-- zebrafish at 5 dpf/2.KEGG/wfs1KOvsWT/ALL/src/dre04622.html]

dre04622


- dre:393144

- Up regulated genes

393144\_2(3.65423823856477)

- dre:561021

- Up regulated genes

561021(2.14284154145004)

- Down regulated genes

561021\_1(-4.68369619674599)

- dre:100148871

- Up regulated genes

100148871(1.46794696008345)

- dre:562552

- Up regulated genes

562552(1.50682290794657)

- dre:564295

- Down regulated genes

564295\_2(-1.27307321576804)

- dre:100002946

- Down regulated genes

100002946(-1.40391573648291)

Close
